# Long-term prediction models for vision-threatening diabetic retinopathy using medical features from data warehouse

**DOI:** 10.1038/s41598-022-12369-0

**Published:** 2022-05-19

**Authors:** Kwanhoon Jo, Dong Jin Chang, Ji Won Min, Young-Sik Yoo, Byul Lyu, Jin Woo Kwon, Jiwon Baek

**Affiliations:** 1 Department of Endocrinology, Incheon St. Mary’s Hospital, College of Medicine, The Catholic University of Korea, Incheon, Republic of Korea; 2 Department of Ophthalmology, Yeouido St. Mary’s Hospital, College of Medicine, The Catholic University of Korea, Seoul, Republic of Korea; 3 Department of Nephrology, Bucheon St. Mary’s Hospital, College of Medicine, The Catholic University of Korea, Gyeonggi-do, Republic of Korea; 4 Department of Ophthalmology, Euijeongbu St. Mary’s Hospital, College of Medicine, The Catholic University of Korea, Gyeonggi-do, Republic of Korea; 5 Department of Ophthalmology, Eunpyeong St. Mary’s Hospital, College of Medicine, The Catholic University of Korea, Seoul, Republic of Korea; 6 Department of Ophthalmology, St. Vincent Hospital, College of Medicine, The Catholic University of Korea, Gyeonggi-do, Republic of Korea; 7 Department of Ophthalmology, Bucheon St. Mary’s Hospital, College of Medicine, The Catholic University of Korea, #327 Sosa-ro, Wonmi-gu, Bucheon, Gyeonggi-do 14647 Republic of Korea; 8 Department of Ophthalmology, College of Medicine, The Catholic University of Korea, Seoul, Republic of Korea

**Keywords:** Health care, Risk factors, Diabetes, Diabetes, Retinal diseases

## Abstract

We sought to evaluate the performance of machine learning prediction models for identifying vision-threatening diabetic retinopathy (VTDR) in patients with type 2 diabetes mellitus using only medical data from data warehouse. This is a multicenter electronic medical records review study. Patients with type 2 diabetes screened for diabetic retinopathy and followed-up for 10 years were included from six referral hospitals sharing same electronic medical record system (n = 9,102). Patient demographics, laboratory results, visual acuities (VAs), and occurrence of VTDR were collected. Prediction models for VTDR were developed using machine learning models. F1 score, accuracy, specificity, and area under the receiver operating characteristic curve (AUC) were analyzed. Machine learning models revealed F1 score, accuracy, specificity, and AUC values of up 0.89, 0.89.0.95, and 0.96 during training. The trained models predicted the occurrence of VTDR at 10-year with F1 score, accuracy, and specificity up to 0.81, 0.70, and 0.66, respectively, on test set. Important predictors included baseline VA, duration of diabetes treatment, serum level of glycated hemoglobin and creatinine, estimated glomerular filtration rate and blood pressure. The models could predict the long-term occurrence of VTDR with fair performance. Although there might be limitation due to lack of funduscopic findings, prediction models trained using medical data can facilitate proper referral of subjects at high risk for VTDR to an ophthalmologist from primary care.

## Introduction

Diabetes mellitus (DM) might be the most important and common metabolic syndrome. Moreover, its prevalence is increasing alongside continued population growth, aging, and escalating rates of obesity^1,2^. Diabetic retinopathy (DR), a significant complication of DM, is the most common cause of newly diagnosed blindness every year, especially in the working-age population^3^.

Progression of DR can lead to vision-threatening DR (VTDR), which is likely to result in vision loss in the absence of treatment^4^. Vision loss in DR is directly associated with clinically significant diabetic macular edema (CSME) and proliferative DR (PDR) and rarely occurs before these complications develop. Therefore, VTDR includes PDR and CSME and is expected to affect 56.3 million people by 2030^5^. With progression of DR to VTDR, the quality of life of patients decreases and the financial burden on society increases^6^. Early diagnosis and proper management of DR can prevent progression.

DM is a general condition, and DR is influenced by systemic factors. The risk factors for incidence and progression of DR have been reported in numerous previous studies and include duration of DM, mean blood glucose level, hemoglobin A1c (HbA1c) level, systolic blood pressure, and presence of nephropathy^4,7–9^. Based on these risk factors, some research has attempted to predict progression of DR using nonlinear methods such as logistic regression and sparse learning^10–13^. Recently, deep learning models for prediction of DR progression using color fundus photography were introduced^14,15^. However, no study to date has predicted the occurrence of VTDR—that is, actual vision loss in DM patients—using advanced machine learning and clinical and laboratory parameters.

Machine learning, an artificial intelligence-based machine learning technology, has shown promising diagnostic performance across specialties including ophthalmology^16,17^. For DR, it has shown promising diagnostic performance using retinal images^17^. Previous research has revealed that DR detected by DL and human graders shares similar risk factors^16^. Electronic medical record (EMR) system has enabled accumulation of enormous data of clinical features including demographics and laboratory tests. In the present study, we assessed the feasibility of a machine learning model trained using medical big data including identified risk factors of DR for prediction of VTDR in a with type 2 DM.

## Results

### Subject characteristics and distribution

Age, ALT, BUN, creatinine, eGFR, glucose, HbA1c, mean VA, low VA, systolic and diastolic BP, and DM treatment duration significantly differed between non-VTDR and VTDR groups (all P ≤ 0.005; Table 1). Male proportion, presence of comorbid CKD, hypertension, cerebrovascular disease, and cardiovascular disease, smoking status, and use of insulin and aspirin also showed difference between non-VTDR and VTDR (all P < 0.001; Table 2).

**Table 1:** Summary of clinical features (continuous variables) of type 2 diabetic patients with and without vision-threatening diabetic retinopathy in datasets of 10-year VTDR prediction.

Clinical features	Non-VTDR (n = 2,924)		VTDR (n = 6,187)		P-value	Missing data (%)
	Mean	SD	Mean	SD		
Age (years)	54.77	11.32	56.69	12.13	< 0.001	0
ALT (IU/L)	25.81	17.62	24.48	18.64	0.002	5.8
AST (IU/L)	23.79	12.10	23.65	14.72	0.659	5.8
BUN (mg/dL)	17.41	9.11	20.10	11.82	< 0.001	9.7
Serum creatinine (mg/dL)	1.09	1.18	1.35	1.52	< 0.001	8.5
eGFR (mL/min/1.73 m2)	75.15	21.54	71.50	28.94	< 0.001	13.2
Serum glucose (mg/dL)	147.98	66.85	170.89	84.72	< 0.001	1.2
HbA1c (%)	7.29	1.48	8.01	1.98	< 0.001	1.3
Height (cm)	158.91	7.71	160.79	8.46	< 0.001	5.5
Weight (kg)	60.56	9.27	61.00	10.04	< 0.001	5.3
BP, diatolic (mmHg)	72.72	9.10	73.36	10.62	0.005	4.1
BP, systolic (mmHg)	131.36	16.09	133.84	19.19	< 0.001	4.1
Low VA (logMAR)	0.80	0.24	0.67	0.29	< 0.001	0.7
Mean VA (logMAR)	0.72	0.26	0.54	0.28	< 0.001	0.7
MAP (mmHg)	111.79	11.96	113.61	14.28	< 0.001	4.1
BMI (kg/m2)	23.98	3.09	23.59	3.20	< 0.001	5.5
Diabetes treatment duration (days)	1334.70	1192.81	1268.80	1395.27	< 0.001	0

*VTDR* vision-threatening diabetic retinopathy, *SD* standard deviation, *ALT* alanine transaminase, *AST* aspartate transaminase, *BUN* blood urea nitrogen, *eGFR* estimated glomerular filtration rate, *HbA1c* glycated hemoglobin, *VA* visual acuity, *BP* blood pressure, *MAP* mean arterial pressure, *BMI* body mass index.

P-value: Independent t-test between non-VTDR and VTDR.

**Table 2:** Summary of clinical features (categorical variables) of type 2 diabetic patients with and without vision-threatening diabetic retinopathy in datasets of 10-year.

Clinical features	Non-VTDR (n = 2,924)	VTDR (n = 6,187)	P-value
Sex, male proportion	46.40	58.10	< 0.001
Chronic kidney disease (%)	22.23	27.58	< 0.001
Hypertension (%)	82.39	73.16	< 0.001
Cerebrovascular disease (%)	30.47	26.04	< 0.001
Cardiovascular disease (%)	33.65	26.87	< 0.001
Smoking (%)	12.10	16.80	< 0.001
Aspirin use (%)	54.86	46.36	< 0.001
Insulin use (%)	60.50	72.47	< 0.001
Clopidogrel use (%)	24.56	24.46	0.919

*VTDR* vision-threatening diabetic retinopathy.

P-value: Chi-square test between non-VTDR and VTDR.

### Performance of the prediction models for VTDR

For 10-year VTDR prediction, F1 score, accuracy, specificity, and AUC values for training were up to 0.661, 0.719, 0.698, and 0.77 by decision tree (fine); 0.666, 0.701, 0.705, and 0.76 by logistic regression; 0.892, 0.892, 0.958, and 0.96 by SVM (fine Gaussian); 0.703, 0.754, 0.725, and 0.74 by naïve Bayes (kernel); 0.806, 0.828, 0.810, and 0.91 by Ensemble decision tree (bagged); and 0.770, 0.795, 0.785, and 0.84 by neural network (wide), respectively (Table 3). The receiver operating characteristic curves for validation is presented in Supplementary Fig. 1. In addition, hyperparameters for optimizable models are presented in Supplementary Table 1.

**Table 3:** Performance parameters of trained model on validation for prediction of VTDR at 10-year.

Methods		Precision	Recall (sensitivity)	F1	Accuracy	Specificity	AUC
**Validation**							
Decision tree	Fine	0.755	0.587	0.661	0.719	0.698	0.77
Logistic regression		0.696	0.639	0.666	0.701	0.705	0.76
SVM	Fine Gaussian	0.834	0.958	0.892	0.892	0.958	0.96
Naïve Bayes	Gaussian	0.705	0.541	0.612	0.680	0.666	0.74
	Kernel	0.713	0.333	0.454	0.626	0.602	0.83
Ensemble decision tree	Boosted tree	0.805	0.625	0.703	0.754	0.725	0.91
	Bagged	0.854	0.762	0.806	0.828	0.810	0.78
	RUSBoosted Tree	0.712	0.651	0.680	0.714	0.716	0.82
Neural network	Narrow	0.798	0.733	0.764	0.789	0.782	0.83
	Wide	0.809	0.735	0.770	0.795	0.785	0.84
	Bilayered	0.758	0.654	0.702	0.741	0.730	0.82
	Trilayered	0.748	0.649	0.695	0.734	0.725	0.80

*VTDR* vision-threatening diabetic retinopathy, *AUC* area under curve of receiver operating characteristics, *SVM* support vector machine.

On the test set, model trained using SVM (fine Gaussian) yielded F1 score, accuracy, and specificity of 0.811, 0.700, and 0.664, respectively (Table 4). When follow-up loss cases were included as no VTDR for sensitivity analysis, sensitivity (recall) and specificity of the models was up to 0.912 and 0.917, respectively (SVM, Table 5). The receiver operating characteristic curves for test set and data set including loss to follow-up is presented in Supplementary Figs. 2 and 3.

**Table 4:** Performance parameters of trainined model on test set for prediction of VTDR at 10-year.

Methods		Precision	Recall (sensitivity)	F1	Accuracy	Specificity
**On test set**						
Decision tree	Fine	0.826	0.556	0.665	0.623	0.455
Logistic regression		0.826	0.627	0.712	0.660	0.487
SVM	Fine Gaussian	0.703	0.958	0.811	0.700	0.664
Naïve Bayes	Gaussian	0.823	0.551	0.660	0.619	0.451
	Kernel	0.914	0.096	0.173	0.386	0.346
Ensemble decision tree	Boosted tree	0.840	0.613	0.709	0.661	0.489
	Bagged	0.797	0.758	0.777	0.707	0.548
	RUSBoosted Tree	0.809	0.636	0.712	0.654	0.480
Neural network	Narrow	0.825	0.672	0.741	0.684	0.513
	Wide	0.762	0.747	0.754	0.673	0.500
	Bilayered	0.815	0.680	0.741	0.681	0.509
	Trilayered	0.810	0.618	0.702	0.646	0.473

*VTDR* vision-threatening diabetic retinopathy, *SVM* support vector machine.

**Table 5:** Performance parameters of trained model on data set including loss to follow-up cases.

Methods		Precision	Recall (sensitivity)	F1	Accuracy	Specificity
**On data set including loss to follow-up cases as no VTDR**						
Decision tree	Fine	0.715	0.813	0.761	0.743	0.780
Logistic regression		0.683	0.679	0.681	0.680	0.676
SVM	Fine Gaussian	0.975	0.912	0.943	0.944	0.917
Naïve Bayes	Gaussian	0.673	0.561	0.612	0.642	0.619
	Kernel	0.784	0.612	0.688	0.720	0.678
Ensemble decision tree	Boosted tree	0.738	0.848	0.789	0.772	0.819
	Bagged	0.910	0.896	0.903	0.903	0.896
	RUSBoosted Tree	0.673	0.738	0.704	0.688	0.706
Neural network	Narrow	0.712	0.731	0.722	0.716	0.720
	Wide	0.762	0.747	0.754	0.673	0.500
	Bilayered	0.708	0.746	0.726	0.717	0.727
	Trilayered	0.703	0.753	0.727	0.716	0.730

*VTDR* vision-threatening diabetic retinopathy, *SVM* support vector machine.

### Important predictors

**Figure 1:**
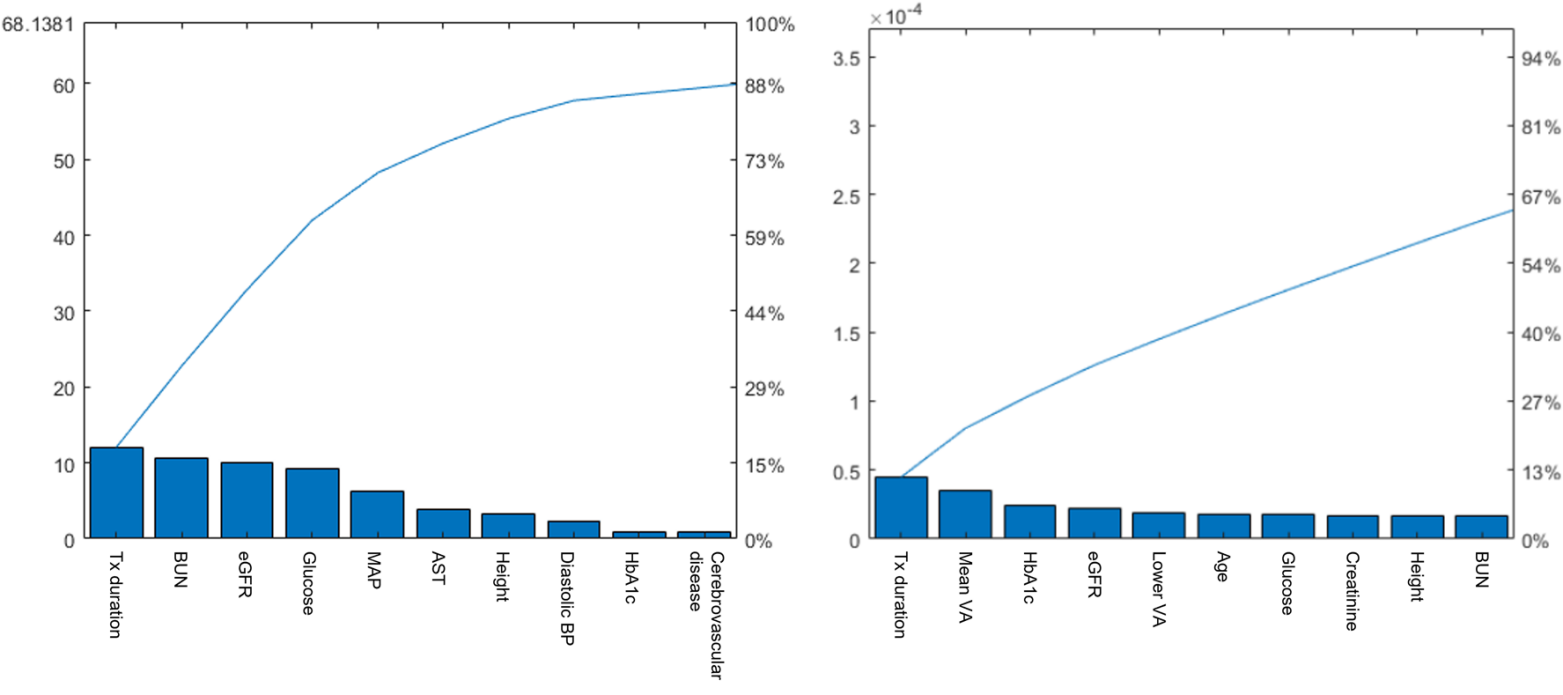
Feature importance analysis. (Left) High-weighted features for VTDR prediction using neighborhood component. (Right) Important predictors revealed by the predictor importance analysis for the bagged ensemble decision tree model.

When neighborhood component analysis using default setting of ‘fscnca’ function of MATLAB in model independent manner, DM treatment duration, BUN, eGFR, glucose, MAP, AST, height, blood pressure, HbA1c, and CVD as features of high weights were revealed as important features (Fig. 1Left). The predictor importance analysis was also performed for bagged ensemble decision tree model. DM treatment duration, baseline VA, HbA1c, sex, eGFR, comorbid hypertension, glucose, creatinine, and height were revealed as predictors of high importance (Fig. 1Right).

## Discussion

The association between clinical features and DR has been studied for many decades^4,18,19^. Based on these results, substantial efforts have been made to predict the incidence and progression of DR in patients with DM. However, there is not much information on prediction of VTDR, a specific state of DR that requires intensive care from ophthalmologists. In the present study, we analyzed the performance of machine learning models in prediction of VTDR using clinical features in patients with type 2 DM.

Previously reported common risk factors for DR in DM patients include duration of DM, age at diagnosis of DM, male gender, smoking, blood glucose, HbA1c, BP, and insulin treatment^4,7–9,19^. Renal function is known to have a close association with DR^20,21^. These known risk factors for DR significantly differed between patient who did and did not develop VTDR in this study. Age was older, male ratio, smoking rate, and BPs was higher, serum levels of glucose, HbA1c, BUN, and eGFR were greater, and insulin use were more frequent in VTDR compared to non-VTDR. BMI was lower in VTDR compared to non-VTDR and similar result had been reported before^22,23^. However, the effect of BMI on DR remain controversial despite various studies and a meta-analysis study revealed negligible effect of BMI on DR^24^.

The proportion of patients with comorbid CKD was higher in the VTDR group. The association between CKD and VTDR has been reported in many previous studies, and retinal microvasculature can provide essential data about concurrent kidney disease status^25^. Progression of retinopathy is reported to be associated with a higher incidence of cardiovascular and cerebrovascular events^26,27^. However, these two conditions were less prevalent in the VTDR group compared to the non-VTDR group in this study. This may be due to characteristics of the study population of the current study which included patients who adhered well to followed-up in referral hospitals for complications of DM. Also, the comorbid cerebrovascular and cardiovascular disease might have been underestimated or underdiagnosed in the VTDR group, and undertreatment of these conditions might have been associated with increased risk of VTDR. More frequent use of aspirin in non-VTDR patients may support this hypothesis.

The prediction model of VTDR was designed to include these known relative features in this study. The best performance was achieved by SVM model. SVM prediction model for VTDR at 10-year using clinical features demonstrated fairly high accuracy, specificity, and AUC. This good result may be explained with a large number of datasets included for training and validation. Since data imbalance during model training was adjusted using ADASYN, sensitivity (recall) was also good despite data imbalance between the VTDR and non-VTDR group during both validation and test. These values were comparable to previous studies cross-sectionally predicted the presence of DR using clinical factors^10,28^. This study has its originality and importance as the models are developed to predict future occurrence of VTDR. Additionally, sensitivity analysis using follow-up loss cases as no-VTDR was performed to overcome selection bias caused by follow-up loss cases. The result revealed high sensitivity and specificity.

Analyses for important predictors revealed eGFR, glucose, blood pressure, HbA1c, and height as features of high importance. These findings are in accordance with those of previous studies by Lui et al.,^28^ who analyzed risk factors of DR and VTDR using logistic regression, and by Oh et al.,^10^ who assessed predicted DR risk using sparse learning. Meanwhile, shorter DM treatment duration was also important in predicting VTDR in this study. This reflect a reasonable fact that compliance of patient in DM control is important factor in future occurrence of VTDR.

There are several limitations to this study. First, there are limitations in prediction performance caused by excluding clinical features with missing data. Also, ophthalmologic history such as previous treatment, surgery, and presence of other conditions that might trigger changes in VA were not investigated, and other known systemic risk factors for DR such as actual duration of DM, alcohol consumption, hematological markers of anemia, hypothyroidism, lipid profile, or genetic profile were not included^4,29^. Most importantly, initial DR state was not available due to the limitation of data warehouse system. Performance of the study models is expected to be improved by including additional clinical features not available in the current study. In addition, the study dataset did not involve patients who were not followed for both DM and DR at the institutions included in this study. DR patients who were followed for DM at outside hospitals or vice versa might have been missed. However, considering that most of DM patients who visit internal medicine department are routinely referred to the ophthalmology departments of all six hospitals, such loss should not be significant. Finally, medical treatment regimen and patient compliance with therapy during follow-up were not considered and can alter the risk of VTDR.

Nonetheless, as the features used for VTDR prediction in this study are easily obtainable from medical records of internists or primary care physicians, the prediction model is expected to be applicable in many clinical settings. The rate of referrals to ophthalmologists by primary care physicians is far below the recommended guidelines, and patients tend to neglect ophthalmologic examinations due to asymptomatic eye status in the earlier stages of DR^30^. We believe these models can be useful in facilitating earlier proper referral of DM patients at high risk for VTDR to ophthalmologists, decreasing rates of vision loss in these patients.

In conclusion, machine learning models using real-world data of demographic and clinical characteristics which did not include funduscopic findings could predict the long-term occurrence of VTDR in patients with type 2 DM. The models can reduce severe vision loss in the DM population by aiding in proper referral of patients at high risk for VTDR to an ophthalmologist.

## Methods

This study was approved by the Institutional Review Board of The Catholic University Medical Center and of each of the involved hospitals (IRB no. XC20WIDI0127): Bucheon St. Mary’s Hospital (Gyeonggi-do, Korea), Incheon St. Mary’s Hospital (Incheon, Korea), Yeoeuido St. Mary’s Hospital (Seoul, Korea), Euijeongbu St. Mary’s Hospital (Gyeonggi-do, Korea), Eunpyeong St. Mary’s Hospital (Seoul, Korea), and St. Vincent’s Hospital (Gyeonggi-do, Korea). The need for written informed consent was waived because of the retrospective design by the Institutional Review Board of The Catholic University Medical Center, and the study was conducted in accordance with the tenets of the Declaration of Helsinki.

### Data preparation

Electronic medical records (EMRs) of subjects diagnosed with type 2 DM and who underwent screening for DR from January 2009 to July 2020 in the ophthalmology department at six university hospitals that share the same EMR system were obtained. In total, a total of 52,927 patients eligible for study inclusion were identified, including 8,180 from Yeoeuido St. Mary’s, 10,185 from Euijeongbu St. Mary’s, 12,356 from Bucheon St. Mary’s, 4,007 from Eunpyeong St. Mary’s, 5,347 from Incheon St. Mary’s, and 12,852 from St. Vincent’s. Of these, 25,878 were male and 27,049 were female.

Diagnosis of type 2 DM was made by internists based on fasting plasma glucose level ≥ 126 mg/dL or two-hour post glucose level ≥ 200 mg/dL after a 75-g oral glucose tolerance test^1^. As VTDR requires treatment, patients with VTDR were identified using diagnosis and treatment code on CDW. Patients with VTDR were defined as those with DR who required intravitreal injection and/or vitrectomy for DR related diagnosis (i.e., CSME, vitreous hemorrhage, proliferative membrane, and/or tractional retinal detachment). Definition for CSME was based on ETDRS criteria and confirmation of hemorrhage, membrane, and retinal detachment was based on pre-operative ophthalmic examination including funduscopic examination, color fundus photography, and optical coherence tomographic images and intraoperative findings observed by surgeons. A subject was classified as VTDR if he returns a VTDR in any period during the follow-up and in any one of both eyes.

### Data cleaning process

**Figure 2:**
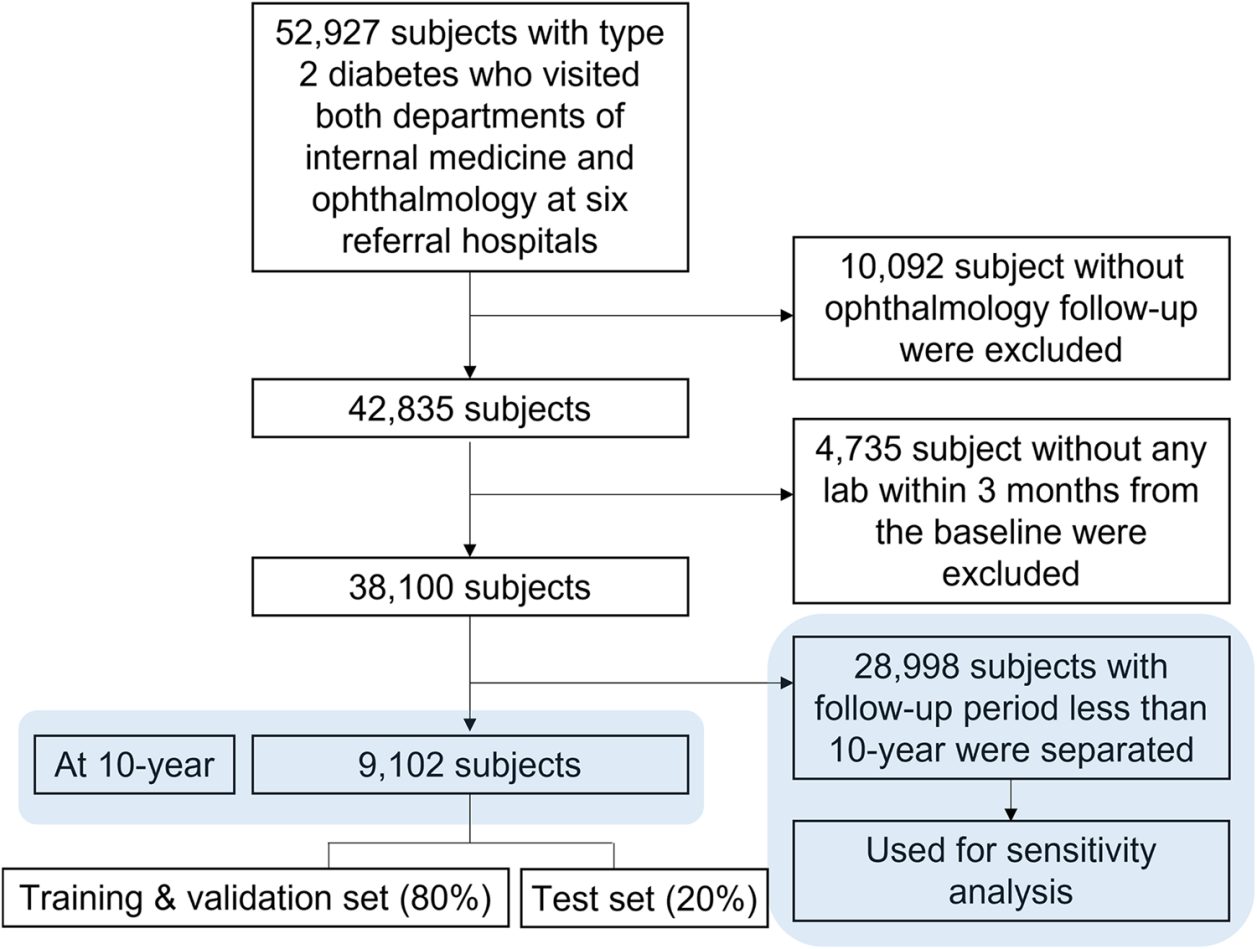
Dataset used in development, validation, and test of diabetic retinopathy risk prediction. This flowchart shows the process of obtaining and cleaning the dataset.

Data standardization and quality control were implemented to ensure data integrity, and exclusion criteria were applied to refine the data used for analysis. Patients screened for DR but who did not follow up at the ophthalmology department were removed (n = 10,092). Then, patients without baseline laboratory data collected within three months from the initial ophthalmologic evaluation (n = 4,735) were removed. In total, data of 38,100 patients were available for the analysis. Models were trained for prediction of VTDR at 10 years from initial DR screening. Study participants followed for at least 10 years totaled 9,102. Remaining 28,998 loss to follow-up data was used for sensitivity analysis (Fig. 2).

Baseline was set as the date of the first ophthalmological screening, while the endpoint was the date of VTDR diagnosis or final follow-up in cases that did not develop VTDR. Medical data at the baseline were obtained from the EMR system. Variables with 20% or more of their values missing were not included in the datasets. Features included in prediction models were as follows. Demographics including age at the first visit, treatment duration of DM, sex, height, weight, systolic and diastolic blood pressure (BP), and smoking status were obtained. Presence of hypertension, chronic kidney disease (CKD), cardiovascular disease, or cerebrovascular disease was collected using diagnostic codes. Use of insulin, aspirin, and clopidogrel was assessed using prescription codes. From laboratory tests, serum levels of alanine aminotransferase (AST), aspartate aminotransferase (ALT), blood urea nitrogen (BUN), creatinine, estimated glomerular filtration rate (eGFR), random glucose, and HbA1c were collected. Only baseline visual acuities (VAs) were available from the ophthalmology chart. Missing data for the remaining variables were handled using regression fitted with supervised machine learning.

### Training and evaluation of the prediction models

All demographic, clinical, and laboratory test features mentioned above were included in model training. The data was divided into training and validation set (80%) and test sets (20%).

Since the 10-year data were imbalanced with higher proportion of VTDR, oversampling of training dataset using adaptive synthetic (ADASYN) sampling algorithm was performed before training^31^ Prediction models were trained for VTDR using decision trees, logistic regression, support vector machine (SVM), naïve Bayes (Gaussian and kernel), and ensemble decision trees (bagged, boosted and RUSboosted). Fifteen-fold cross-validation was used during training and validation of models. Hyperparameters were optimized automatically using optimizable training options for each model of ‘Classification Learner’ app on MATLAB (MathWorks, Inc., Natick, MA, USA). For neural network, one fully connected layer sized of 10 (wide), 100 (narrow) and two- and three-fully connected layer size of 10 were used for training. Then, trained models were validated on original data set and tested on test set. The performance of models was evaluated using accuracy, specificity, F1 score, receiver operating characteristics, and area under the curve (AUC). F1 Score was calculated as 2 x ((precision x recall) / (precision + recall)). All experiments were performed using MATLAB 2021a.

### Statistics

Statistical analysis was performed using MATLAB 2021a. T-tests were used to compare demographics between groups. Chi-square test was used to compare categorical variables. Accuracy, precision, recall, specificity, and F1 scores were calculated for each model. The F1 score was calculated as 2 × (precision) × (recall) / [(precision) + (recall)]. Continuous variables are presented as mean ± standard deviation.

## Supplementary Information

Supplementary Legends.

Supplementary Figure 1.

Supplementary Figure 2.

Supplementary Figure 3.

Supplementary Table 1.

## Data Availability

The datasets generated and/or analyzed during the current study are available from the corresponding author upon request.
