# Author Correction: Long-term prediction models for vision-threatening diabetic retinopathy using medical features from data warehouse

**DOI:** 10.1038/s41598-022-18012-2

**Published:** 2022-08-05

**Authors:** Kwanhoon Jo, Dong Jin Chang, Ji Won Min, Young-Sik Yoo, Byul Lyu, Jin Woo Kwon, Jiwon Baek

**Affiliations:** 1 Department of Endocrinology, Incheon St. Mary’s Hospital, College of Medicine, The Catholic University of Korea, Incheon, Republic of Korea; 2 Department of Ophthalmology, Yeouido St. Mary’s Hospital, College of Medicine, The Catholic University of Korea, Seoul, Republic of Korea; 3 Department of Nephrology, Bucheon St. Mary’s Hospital, College of Medicine, The Catholic University of Korea, Gyeonggi-do, Republic of Korea; 4 Department of Ophthalmology, Euijeongbu St. Mary’s Hospital, College of Medicine, The Catholic University of Korea, Gyeonggi-do, Republic of Korea; 5 Department of Ophthalmology, Eunpyeong St. Mary’s Hospital, College of Medicine, The Catholic University of Korea, Seoul, Republic of Korea; 6 Department of Ophthalmology, St. Vincent Hospital, College of Medicine, The Catholic University of Korea, Gyeonggi-do, Republic of Korea; 7 Department of Ophthalmology, Bucheon St. Mary’s Hospital, College of Medicine, The Catholic University of Korea, #327 Sosa-ro, Wonmi-gu, Bucheon, Gyeonggi-do 14647 Republic of Korea; 8 Department of Ophthalmology, College of Medicine, The Catholic University of Korea, Seoul, Republic of Korea

Correction to: *Scientific Reports* 10.1038/s41598-022-12369-0, published online 19 May 2022

In the original version of this Article Kwanhoon Jo was incorrectly affiliated with ‘Department of Ophthalmology, Yeouido St. Mary’s Hospital, College of Medicine, The Catholic University of Korea, Seoul, Republic of Korea’. Additionally, Dong Jin Chang was incorrectly affiliated with ‘Department of Endocrinology, Incheon St. Mary’s Hospital, College of Medicine, The Catholic University of Korea, Incheon, Republic of Korea’.

The correct affiliations are listed below.

**Kwanhoon Jo**

Department of Endocrinology, Incheon St. Mary’s Hospital, College of Medicine, The Catholic University of Korea, Incheon, Republic of Korea.

**Dong Jin Chang**

Department of Ophthalmology, Yeouido St. Mary’s Hospital, College of Medicine, The Catholic University of Korea, Seoul, Republic of Korea.

The original Article has been corrected.

