# Supplementary material for: Long-term prediction models for vision-threatening diabetic retinopathy using medical features from data warehouse
Source: Sci Rep. 2022 May 19;12:8476. doi: 10.1038/s41598-022-12369-0 (PMC9119940; doi:10.1038/s41598-022-12369-0)
Supplement: Supplementary file 1 — Supplementary Legends. [file 41598_2022_12369_MOESM1_ESM.docx]

**Supplementary Figure 1. Receiver operating characteristic curves for validation of each model.**

(A) Fine decision tree model (B) Fine Gaussian support vector machine model (C) Logistic regression model (D) naïve Bayes model (E) Optimized bagged ensemble model.

**Supplementary Figure 2. Receiver operating characteristic curves for trained models on test set for prediction of VTDR at 10-year.**

(A) Fine decision tree model (B) Fine Gaussian support vector machine model (C) Logistic regression model (D) naïve Bayes model (E) Optimized bagged ensemble model.

**Supplementary Figure 3. Receiver operating characteristic curves for trained models on data set including loss to follow-up cases.**

(A) Fine decision tree model (B) Fine Gaussian support vector machine model (C) Logistic regression model (D) naïve Bayes model (E) Optimized bagged ensemble model.
