# Supplementary figures and images for: Long-term prediction models for vision-threatening diabetic retinopathy using medical features from data warehouse
Source: Sci Rep. 2022 May 19;12:8476. doi: 10.1038/s41598-022-12369-0 (PMC9119940; doi:10.1038/s41598-022-12369-0)

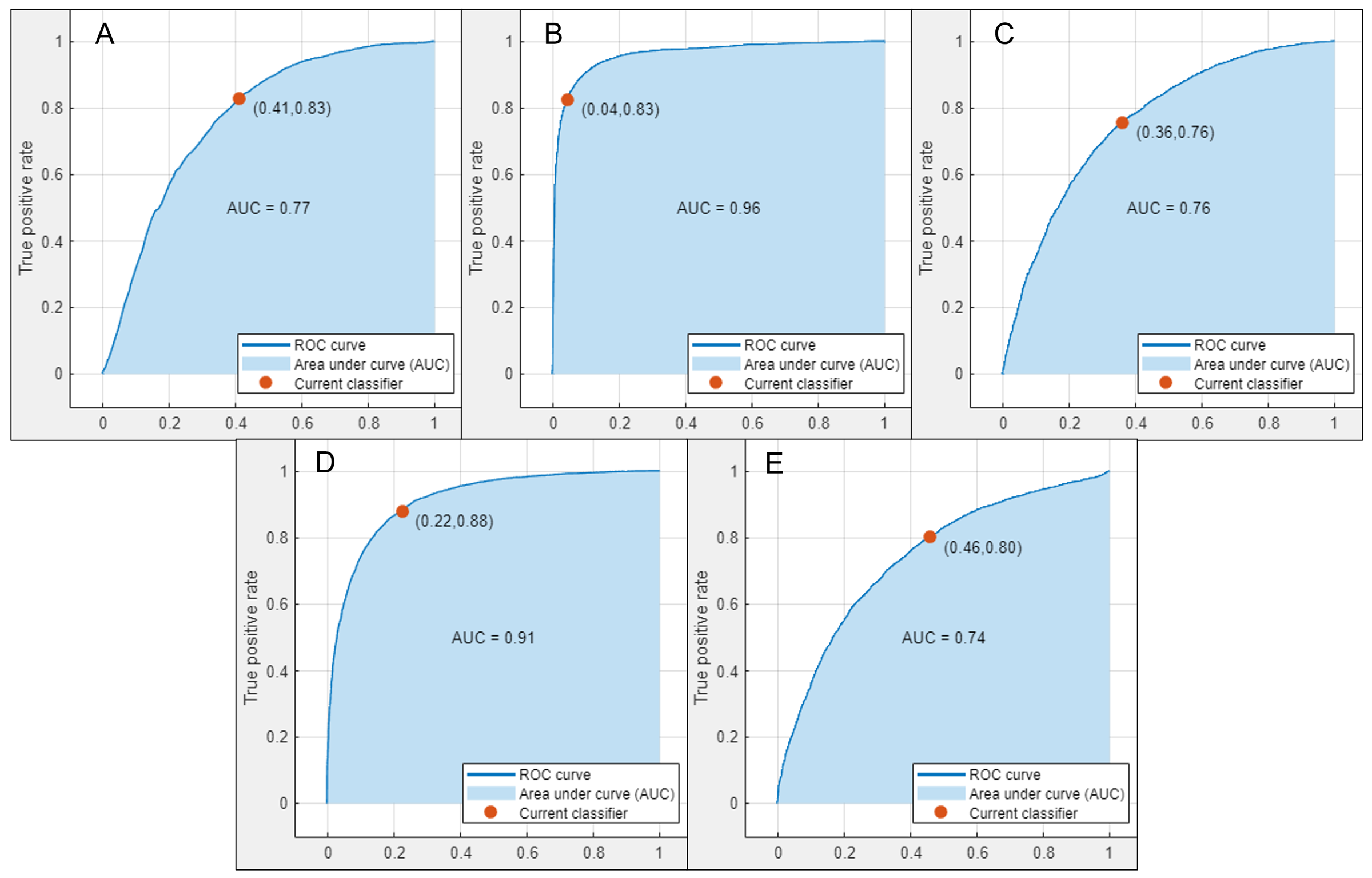

Supplement: Supplementary file 2 — Supplementary Figure 1. [file 41598_2022_12369_MOESM2_ESM.tif]

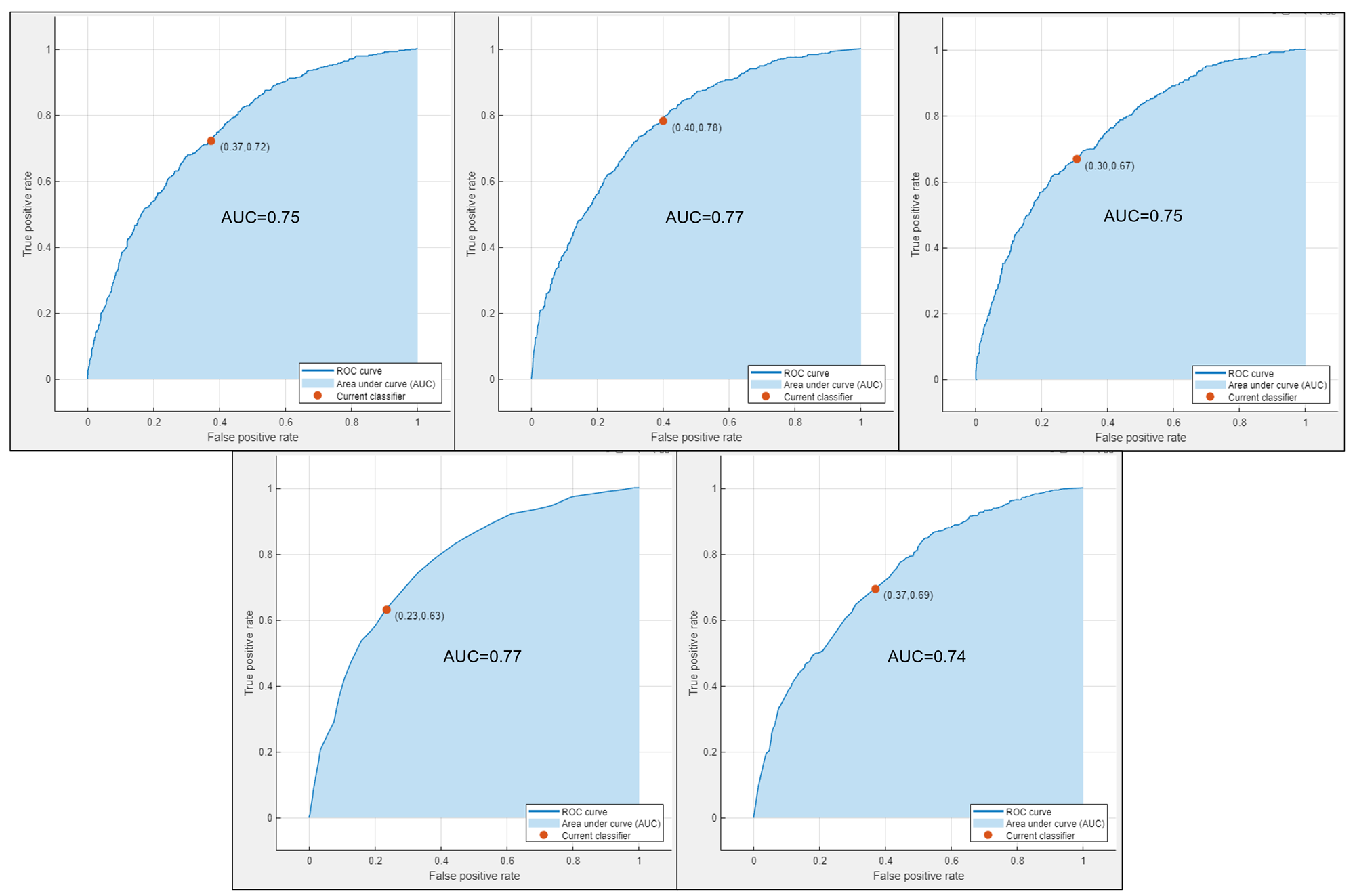

Supplement: Supplementary file 3 — Supplementary Figure 2. [file 41598_2022_12369_MOESM3_ESM.tif]

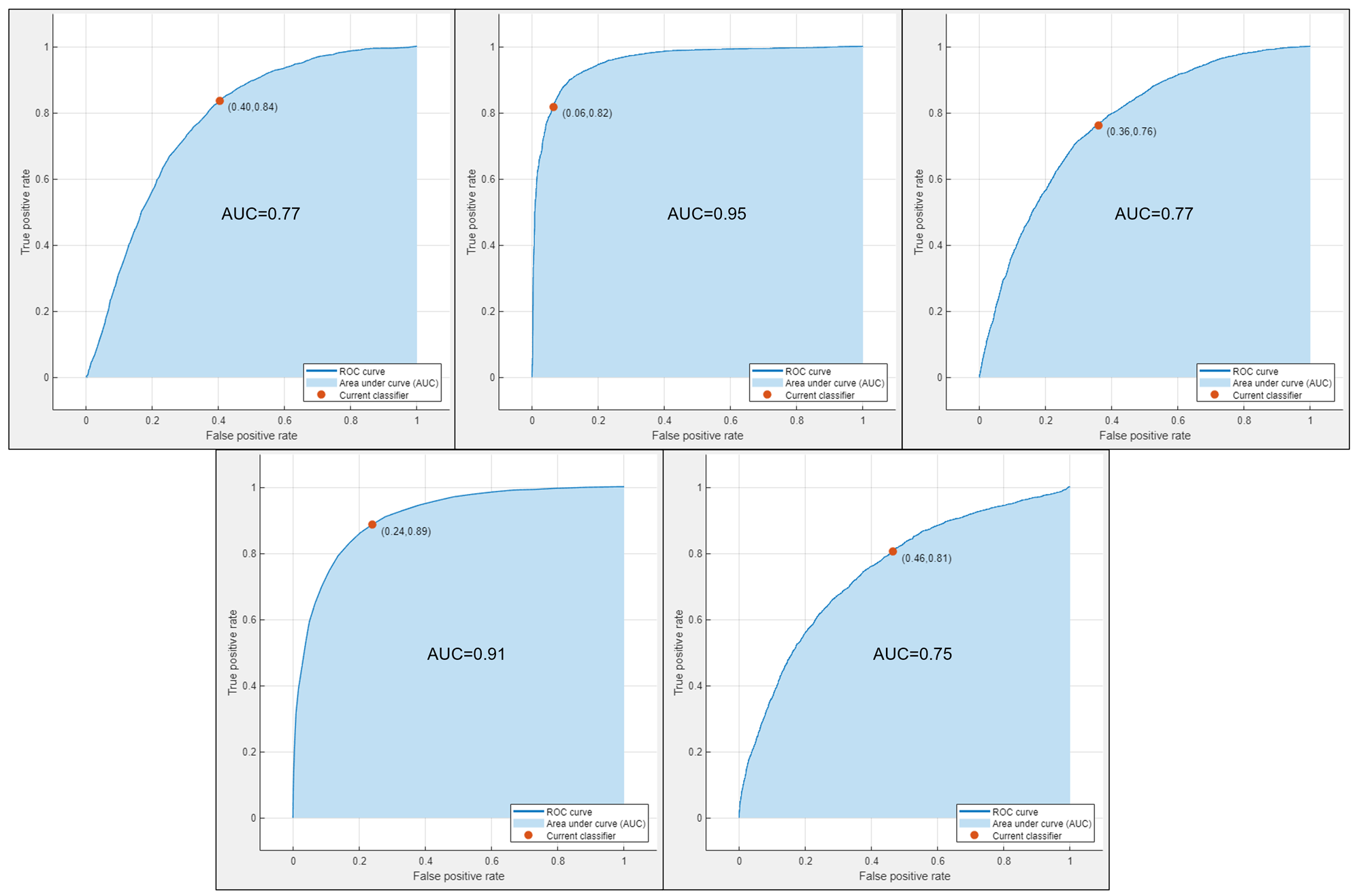

Supplement: Supplementary file 4 — Supplementary Figure 3. [file 41598_2022_12369_MOESM4_ESM.tif]
