# Supplementary material for: Long-term prediction models for vision-threatening diabetic retinopathy using medical features from data warehouse
Source: Sci Rep. 2022 May 19;12:8476. doi: 10.1038/s41598-022-12369-0 (PMC9119940; doi:10.1038/s41598-022-12369-0)
Supplement: Supplementary file 5 — Supplementary Table 1. [file 41598_2022_12369_MOESM5_ESM.docx]

| Supplementary Table 1. Optimized hyperparameters of trainined models for prediction of VTDR at 10-year. | |
| --- | --- |
| Methods | Optimized hyperparameters |
| Decision tree | Maximum number of splits: 702 Split criterion: Maximum deviance reduction |
| SVM | Kernel function: Gaussian Box constraint level: 38.432 Standarize data: true |
| Naïve Bayes | Distribution name: Gaussian |
| Ensemble decision tree | Ensemble method: RUSBoost Number of learners: 95 Learning rate: 0.0010354 Maximum number of splits: 10193 |
| Neural network | Number of fully connected layers: 3 Activation: Than Iteration limite:1000 Regularization strength (Lambda): 0.00019572 Standardize data: true |
| VTDR: vision-threatening diabetic retinopathy; SVM: support vector machine. | |
